# Synthesis, *In Vitro* and *In Vivo* Evaluation of the *N*-ethoxycarbonylmorpholine Ester of Diclofenac as a Prodrug

**DOI:** 10.3390/ph7040453

**Published:** 2014-04-14

**Authors:** Jamal A. Jilani, Nasir M. Idkaidek, Karem H. Alzoubi

**Affiliations:** 1 Department of Medicinal Chemistry and Pharmacognosy, Faculty of Pharmacy, Jordan University of Science and Technology, P.O. box 3030, Irbid 22110, Jordan; 2 Faculty of Pharmacy, University of Petra, Amman 11196, Jordan; E-Mail: nidkaidek@uop.edu.jo; 3 Department of Clinical Pharmacy, Jordan University of Science and Technology, P.O. box 3030, Irbid 22110, Jordan; E-Mail: khalzoubi@just.edu.jo

**Keywords:** diclofenac, prodrug, ethoxycarbonylmorpholine, pharmacokinetics, ulcerogenicity, prodrug hydrolysis

## Abstract

The *N*-ethoxycarbonylmorpholine moiety was evaluated as a novel prodrug moiety for carboxylic acid containing drugs represented by diclofenac (**1**). Compound 2, the *N*-ethoxycarbonylmorpholine ester of diclofenac was synthesized and evaluated as a potential prodrug. The stability of the synthesized prodrug was evaluated in solutions of pH 1 and 7.4, and in plasma. The ester’s half lives were found to be 8 h, 47 h and 21 min in pH 1, pH 7.4 and plasma, respectively. Equimolar doses of diclofenac sodium and its synthesized prodrug were administered orally to a group of rabbits in a crossover study to evaluate their pharmacokinetic parameters. The prodrug **2** shows a similar rate and extent of absorption as the parent drug (**1**). The ulcerogenicity of the prepared prodrug was evaluated and compared with the parent drug. The prodrug showed less ulcerogenicity as detected by fewer number and smaller size of ulcers. In conclusion, the newly synthesized *N*-ethoxycarbonylmorpholine ester of diclofenac prodrug showed appropriate stability properties at different pHs, similar pharmacokinetic profile, and much less ulcerogenecity at the GIT compared to the parent drug diclofenac.

## 1. Introduction

**Figure 1:**
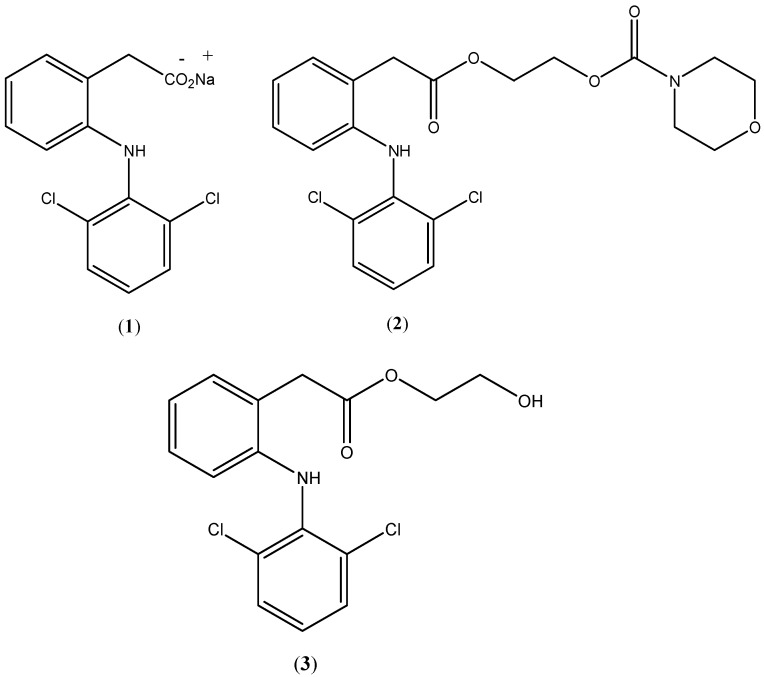
Chemical structures of diclofenac sodium (**1**), prodrug (**2**) and hydroxyethyldiclofenac (**3**).

Diclofenac (**1**; Figure 1) and its different salts are well-known nonsteroidal anti-inflammatory drugs (NSAIDs) which are largely used orally, topically and parenterally to treat a wide range of inflammation conditions [1]. As with many NSAIDs, prolonged use of diclofenac leads to unwanted side-effects [2], the most dangerous among which is gastrointestinal ulcerations and bleeding [1,2]. Extensive research efforts have been done to overcome this serious side-effect. This resulted in the discovery of selective cyclooxygenase 2 (Cox-2) inhibitors, which were safer concerning the gastrointestinal tract (GIT) side-effects [3], however, their cardiotoxic effects have limited their use leaving the door open for solutions to the problematic NSAID side-effects [4].

The use of prodrugs is a useful approach to minimize the GIT side effects of NSAIDs [3,5,6,7]. The chemical structures of the prodrug moieties determine the physicochemical properties of the prodrug under development, thus, affect its pharmacological profile. In general, selecting prodrug moieties for NSAIDs represents a crucial factor to achieve prodrug development objectives including better pharmacokinetic properties and no safety issues [8,9]. In the present work, a novel prodrug promoiety, namely *N*-ethoxycarbonylmorpholine, was selected to temporarily mask the carboxylate moiety of diclofenac. The aim of this prodrug was to reduce GIT side effects while maintaining similar pharmacokinetic parameters to those of the parent compound. It is expected that the proposed prodrug, namely, the *N*-ethoxycarbonylmorpholine ester of diclofenac (**2**; Figure 1) will be susceptible to plasma esterases and sufficiently stable to withstand conditions of the GIT, while producing fewer adverse GIT effects.

## 2. Experimental

Diclofenac sodium was obtained from the Hikma Pharmaceutical Company (Amman, Jordan). All chemical reagents were purchased from commercially available sources. Melting points were taken on a Gallenkamp capillary melting point apparatus (model MPD 350.BM 2.5, Kent, UK). ^1^H-NMR spectra were recorded on a Bruker Avance 400 MHz instrument (Bruker AXS GmbH, Karlsruhe, Germany), and chemical shifts (δ) are reported as parts per million (ppm) relative to internal standard, tetramethylsilane. Infrared spectra were obtained with a Nicolet Impact 410 (Markham, ON, Canada). Mass spectroscopy data were obtained by VG7070 mass spectrometer (M-Scan Inc. West Chester, PA, USA). 2-Hydroxyethyldiclofenac (**3**) was prepared according to a previously described method [10].

*N-[(2-Bromoethoxy)carbonyl]morpholine* (**4**). To a mixture of morpholine (5 g, 0.058 mol), benzene (20 mL) and pyridine (9 g) 2-bromoethyl-chloroformate (11 g, 0.059 mol) was added. The reaction mixture was heated under reflux for 8 h. The solid was removed by filtration. The filtrate was evaporated yielding an oily material, which was allowed to cool to room temperature. Distillation under vacuum gave pure **4** (10.1 gm, 73%), which solidified upon cooling. mp 42–44 °C, IR (cm^−1^) 1,700; ^1^H-NMR (CDCl_3_) δ 3.4 (m, 4Hs, CH_2_-N-CH_2_); 3.6 (t, 2H, CH_2_Br, *J* = 6 Hz), 3.7 ( m, 4Hs, CH_2_OCH_2_), 4,4 (t, 2Hs, CH_2_OCO, *J* = 6 Hz); MS (m/e) 238 M^+^.

*N-Ethoxycarbonylmorpholine ester of 2-[(2,6-dichlorophenyl]) amino] benzeneacetic acid* (**2**). Compound **4** (2.25 g, 9.4 mmol) was added to a solution of 2-[(2,6-dichloro-phenyl])amino]benzeneacetic acid sodium salt (diclofenac sodium, 3 g, 9.4 mmol) in DMF (5 mL). The mixture was stirred for 8 h at 60 °C. The reaction was cooled to room temperature and 20 mL ethyl acetate was added to the reaction mixture. The resulting mixture was filtered and the filtrate was extracted twice with 25 mL water then dried over anhydrous MgSO_4_. Evaporation of the ethyl acetate yielded an oily liquid, which was solidified upon standing. Recrystallisation from methanol gave 2.95 gm of **2** (69%); mp 57–58 °C; IR (cm^−1^) C=O at 1,703, 1,706; ^1^H-NMR δ in CDCl_3_: 3.2–3.7 (m, 4H, CH_2_NCH_2_ ), 3.85 (s, 2H, benzylic CH_2_), 4.25–4.40 (m, 8H, (CH_2_OCH_2_)_2_), 6.9–7.4 (m, 7 H, aromatic); MS (*m/e*) 435 (M^+^).

### 2.1. High Performance Liquid Chromatography (HPLC) Method

**Figure 2:**
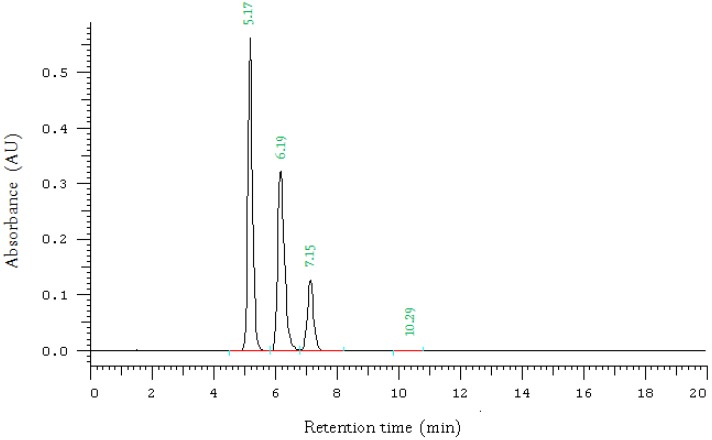
HPLC chromotagram of diclofenac sodium (**1**), the *N*-ethoxycarbonylmorpholine ester prodrug (**2**), and 2-hydroxyethyldiclofenac (**3**) showing retention times of 5.17 min 6.19 min and 7.15 min, respectively.

The HPLC analysis was performed using LaChrom Merck Hitachi system that consists of a D-7000 interface, L-7200 diode array detector, pump L-7150 and D-7000HSM software (Schaumburg, IL, USA). The UV detector was set at 280 nm wave length. The HPLC column was an Agela unisol-C18, 5 micron 4.6 × 150. Mobile phase was 80% methanol, 20% water. To each litre, 2 mL acetic acid were added. The mobile phase was pumped at flow rate 1.5 mL/min. The retention times were: 5.17 min 6.19 min and 7.15 min for 2-hydroxyethyldiclofenac, diclofenac sodium and the morpholine prodrug, respectively (Figure 2). The analytical method was fully validated.

### 2.2. Hydrolysis Kinetics in Aqueous Buffer Solution

The prodrug under investigation was incubated in 0.1 N HCl (pH 1.0) and phosphate buffer (pH 7.4). The formed solutions were kept in a shaking water bath at about 37 °C for eight h. At appropriate intervals, samples (100 µL) were analyzed using the HPLC method described earlier.

### 2.3. Hydrolysis Kinetics in Human Plasma

The prodrug under investigations was incubated in human plasma using a shaking water bath, which was set at 37 ± 0.3 °C. An amount of 25 µL of 1.0 mg/mL stock solution of the prodrug under investigation was added to 5 mL of the incubated plasma. At appropriate time points, samples of 100 μL were withdrawn and deproteinized by adding equal volumes of acetonitrile. The formed mixture was mixed on a vortex then centrifuge at 2,500 rpm for 10 min. The clear supernatant was analyzed using the HPLC method.

### 2.4. In Vivo Evaluation

The *in vivo* pharmacokinetic and ulcerogenicity assessment were carried out according to international guidelines and according to the ACUC Guidelines adopted by the Jordan University of Science and Technology.

### 2.5. In Vivo Comparative Bioavailability Study

To assess the *in vivo* behavior of prodrug under testing, an open label randomized two-way cross over design using twelve healthy rabbits was adopted. Each rabbit received 5 mg of diclofenac sodium (**1**) solution and 7 mg (equimolar dose) of the prodrug **2** as an aqueous suspension according to the randomization plan. No food intake was allowed 12 h before drug administration. The washout period was 7 days. Collection of blood samples were carried out according to the clinical protocol. Blood samples (1 mL) were taken for drug analysis at 0.33, 0.66, 1.00, 1.5, 2.00, 2.5, 3.00, 4.00, 5.00, 6.00 and 8.00 h after administration of drugs under study. A blank sample was also taken before dosing. For blood collection, an infant catheter was placed into the rabbit’s ear vein. Blood samples were collected into 1.5 mL Eppendrof tubes, shacked and centrifuged at 4000 rpm for 10 min. The label on the blood collection tubes indicated animal number, study phase and the designated sample number. After centrifugation, plasma was stored immediately in a −20 °C freezer.

### 2.6. In Vivo Ulcerogenicity Study

The ulceroginicity of diclofenac sodium and its prepared prodrug **2** were evaluated. Three groups (six animals each) of male Sprauge-Dawley rats (150–200 gm) were fasted for 8 h prior to administration of single equimolar doses of diclofenac sodium (100 mg/kg), its prodrug (140 mg/kg), or vehicle. Free access to food and water were provided throughout the experiment. The rats were sacrificed under ether anesthesia after 17 h. The stomach of the rats was dissected out of the body, and stomach content was emptied and washed with saline. The stomach was then opened and wiped with a clean swap dipped in saline. The degree of mucosal damage was assessed using a 4× binocular magnifier. The severity of the damage was assessed according to a scoring system where the presence of <5 punctiforms with no ulcers was given the score of zero. A punctiform was defined as a lesion of less than 1 mm. If >5 punctiforms were present with no ulcer, a score of 1 was given. If 1–5 small ulcers (1–2 mm in diameter) were observed, a score of 2 was given. If >5 small ulcers or one large ulcer (>2 mm in diameter) was present, a score of 5 was given. If >1 large ulcer was observed, a score of 4 was given. The scores were averaged and the mean score was tabulated as ulcerogenicity index for the drugs under investigation.

### 2.7. Data Analysis

Pharmakokinetic parameters (AUC_0-t_, AUC_0-inf_, half-life, elimination rate constant, C_max_ and T _max_) were determined by standard non-compartmental analysis and then subjected to statistical analysis of variance, un-paired t-test, and Kruskal-Wallis test using the Kinetica 2000^®^ program version 3.0 by Innaphase Corporation (Philadelphia, PA, USA). Bioavailability conclusions were based on AUC_0-t_, AUC_0-inf_, and C_max_ parameters. The elimination phase was unclear in some rabbits and hence AUC_0-inf_ and elimination parameters were not calculated and, thus, not used in bioavailability calculation. Comparative bioavailability extent was done using the equation: F = AUCt_prodrug_/AUCt_drug_.

## 3. Results and Discussion

**Scheme 1:**
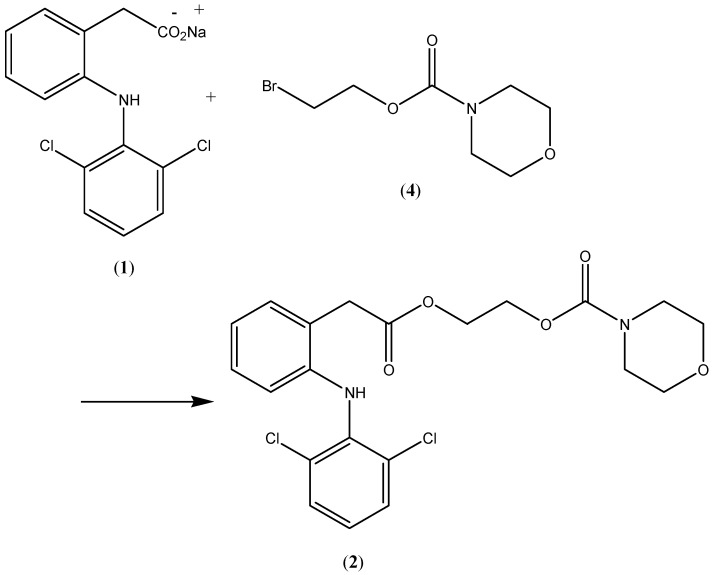
Synthesis of the prodrug **2**.

**Scheme 2:**
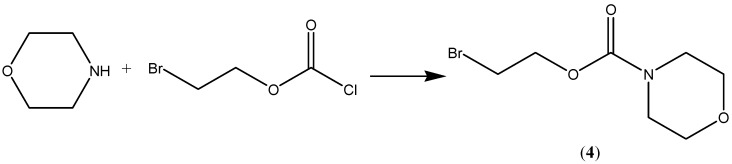
Synthesis of compound **4**.

Compound 2, the *N*-ethoxycarbonylmorpholine ester of diclofenac, was prepared by reacting *N*-[(2-bromoethoxy)carbonyl] morpholine (**4**) with the sodium salt of diclofenac as shown in Scheme 1. The key intermediate *N*-morpholinecarbonyloxy-2-ethyl bromide was prepared by reacting morpholine with commercially available 2-bromoethylchloroformate (Scheme 2). The possible metabolic intermediate, 2-hydroxyethyldiclofenac (**3**) was prepared by reacting diclofenac sodium with 2-bromoethanol according to a previously mentioned procedures [10]. The structure of each of the prepared compounds was confirmed using different spectroscopic techniques including ^1^H-NMR, IR and MS.

Stability studies Showed that the half lives of the prodrug were of 8 h, 47 h and 21 min in pH 1, 7.4 and in plasma, respectively. Additionally, only the parent drug diclofenac (**1**) appeared, as a result of hydrolysis, and none of the other possible intermediate **3** was observed. 

**Figure 3:**
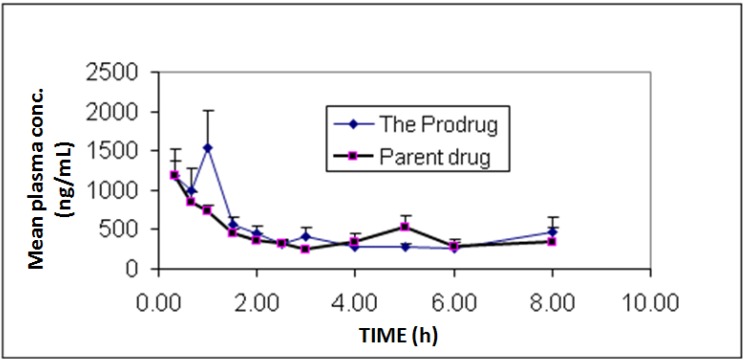
Mean plasma concentrations profile of diclofenac sodium (**1**) and diclofenac produced from the hydrolysis of the *N*-ethoxycarbonylmorpholine ester prodrug (**2**). Values are mean ± SEM from 6 rabbits.

*In vivo* bioavailability and pharmacokinetics experiments showed individual and mean plasma concentrations of diclofenac and its prodrug as presented in Figure 3. Additionally, pharmacokinetic parameters are summarized in Table 1. The study showed similar bioavailability (F = 1.05) for diclofenac prodrug compared to diclofenac sodium.

**Table 1:** Pharmacokinetic parameters of the parent drug diclofenac (**1**) *versus* its *N*-ethoxycarbonylmorpholine ester prodrug (**2**) in rabbits (*n* = 6/group). Both drugs were administered orally at a dose of 5 mg/kg for diclofenac, and 7 mg (equimolar dose) of the prodrug (**2**). None of the pharmacokinetic parameters of the prodrug (**2**) was different from the corresponding values of the parent drug (*p* > 0.05; using un-paired *t*-test).

	AUC_0-t_	* AUC_0-inf_	C_max_	T_max_	T _1/2_	K_el_	AUC_0-t_/AUC_inf_	K_el_ start	K_el_ stop
	(ng h/mL)	(ng h/mL)	(ng/mL)	(h)	(h)	(1/h)		(h)	(h)
Diclofenac sodium (1)									
MEAN	3188.33	3,420.51	1,220.8	0.61	1.69	0.45	89.38	4.40	7.20
SD	1622.77	2,247.09	383.37	0.68	0.67	0.16	7.31	1.52	1.79
SEM	662.49	1,123.54	156.51	0.28	0.33	0.08	3.65	0.68	0.80
CV%	50.90	65.69	31.40	112.0	39.57	34.3	8.17	34.47	24.85
*N*-ethoxycarbonylmorpholine ester prodrug (2)									
MEAN	3350.20	2,796.70	1,807.03	1.08	1.41	0.72	92.81	3.00	6.13
SD	1963.01	1,334.75	1,537.47	0.59	0.88	0.58	6.43	1.47	2.59
SEM	801.39	667.37	627.67	0.24	0.44	0.29	3.21	0.74	1.30
CV%	58.59	47.73	85.08	54.86	61.95	80.5	6.93	49.07	42.35

* Since some profiles did not show clear elimination and hence did not have AUC infinity data, the average AUC infinity for the prodrug was smaller than average AUC_0-t_.

With an aim of minimizing GIT side effects utilizing the prodrug approach, a comparative ulcerogenicity evaluation was done via single dose administration to three groups of rats of diclofenac sodium (**1**), the prepared prodrug **2**, or placebo (no drug). Group 1, which received no treatment, and no lesion was observed, received ulcer score of zero. Group 2, which received diclofenac sodium, and induced an average of 82 lesions; six of them were larger than 2 mm, received an ulcer score of four. The third group, which received diclofenac prodrug (**2**), and showed less than five punctiform lesions, got an ulcer score of zero.

This study showed the potential use of *N*-ethoxycarbonylmorpholine group as a novel prodrug moiety for carboxylic acid-containing drugs represented by diclofenac. The study shows that masking the carboxylic moiety of diclofenac with the above mentioned moiety, maintains the pharmacokinetic parameters of the parent compound, while it reduces its adverse effect. Previous studies have reported other forms of diclofenac prodrugs with lower GI adverse effects, or with properties that aid delivery of the drug to certain distal body organs. For example, 1-(2,6-dichlorophenyl)indolin-2-one is a diclofenac prodrug which demonstrated relevant anti-inflammatory properties without GI ulceration effect [11,12]. Diclofenac ester prodrugs were found to be potent anti-inflammatory drugs with less ulcerogenic potential than the parent diclofenac sodium [13,14,15,16,17]. Additionally, Conjugation of diclofenac with 2-hydroxylethylmethacrylate resulted in a monomeric drug derivative of diclofenac that possesses lower ulcergenic properties than the parent drug [18]. Moreover, a novel bisphosphonic prodrug of diclofenac was used for osteotropic delivery of diclofenac [19,20]. The current study confirms the use of prodrug approach of previous studies; yet, it offers a novel prodrug moiety, namely, *N*-carbonyloxymorpholine promoeity, which reduced the GI toxicity of the parent drug diclofenac, while maintained its pharmacokinetic parameters. This novel prodrug promoiety could be applied to other carboxylic acid-containing drugs.

In order to achieve the objective of this work, the developed prodrug should withstand the chemical conditions encountered in the GIT by being absorbed intact and then to undergo hydrolysis yielding the parent compound. This is reflected in the obtained results showing that half lives of the prodrug were 8 h at pH 1, 47 h at pH 7.4, and only 21 min at the plasma. This result reflects sufficient stability in GIT that would ensure intact absorption. At the same time, the plasma study shows enzymatic susceptibility to plasma esterase that would insure complete delivery of the parent compound as required after absorption. The stability study showed peaks for only the parent drug diclofenac (**1**) with no appearance of the other possible intermediate **3**. Therefore, it seems that only the aliphatic ester site actually undergoes hydrolysis despite the presence of two possible sites susceptible to hydrolysis. This observation is expected because of the much slower rate of hydrolysis of carbamates compared to aliphatic esters, where carbamates in general are poor substrate for mammalian esterases [21,22].

The prodrug **2** synthesized in this study maintained the pharmacokinetic profile of the parent drug as indicated by a similar bioavailability and rate of absorption of the prodrug and the parent compound. However, a double peak phenomenon was observed in some rabbits. This is suggested to be due to multiple segment absorption in the rabbit gut and/or due to prodrug degradation in the small intestine and subsequent absorption of the parent drug at a higher rate. Future work can be directed towards performing thorough *in vitro* solubility and stability testing in gut environment for the prodrug to enhance its bioavailability.

Current results indicate that the newly synthesized *N*-ethoxycarbonylmorpholine ester of diclofenac (compound **2**) possesses lower GIT ulcerogenecity. This result supports previous findings that masking the carboxylic acid moiety of NSAIDs gives safer compounds [5,11].

## 4. Conclusions

In conclusion, the *N*-ethoxycarbonylmorpholine group may represent a useful approach for masking carboxylate moieties of drugs. The newly synthesized *N*-ethoxycarbonylmorpholine ester of diclofenac (compound **2**) showed success in reducing the adverse GIT effects of diclofenac, while maintaining its pharmacokinetic profile. Future work could be directed toward manipulating the structure of this moiety in an effort to obtain a better pharmacokinetic profile than the parent drug. Branching of the ethylene spacer as well as increasing its length could be used as tools to modify the behaviour of the prodrug in terms of stability and pharmacokinetics.
